# Evaluating Orally Administered Meloxicam-Loaded Polymeric Nanocapsules in Female Dogs: A Population Pharmacokinetic Modeling Study

**DOI:** 10.3390/ph19030412

**Published:** 2026-03-03

**Authors:** Flávia Elizabete Guerra Teixeira, Graziela de Araújo Lock, Renata Giacomeli, Camila de Oliveira Pacheco, Tamara Ramos Maciel, Ana Pozzato Funghetto-Ribeiro, Gabriela Lugoch, Diego Vilibaldo Beckmann, Marília Teresa de Oliveira, Sandra Elisa Haas

**Affiliations:** 1 Pharmacology and Pharmacometric Laboratory, LABFAR, Federal University of Pampa (UNIPAMPA), Uruguaiana 97501-970, RS, Brazil; flaviateixeira.aluno@unipampa.edu.br (F.E.G.T.); graziela.lock@gmail.com (G.d.A.L.); rennatinha_giacomeli@hotmail.com (R.G.); coliveirapacheco@gmail.com (C.d.O.P.); tamararmaciel@gmail.com (T.R.M.); acfunguetto@gmail.com (A.P.F.-R.); 2 Pharmaceutical Sciences Post Graduate Program, Federal University of Pampa (UNIPAMPA), Uruguaiana 97501-970, RS, Brazil; 3 Animal Science Post Graduate Program, Federal University of Pampa (UNIPAMPA), Uruguaiana 97501-970, RS, Brazil; gabrielalugoch54@hotmail.com (G.L.); dvbeckmann@hotmail.com (D.V.B.); mariliaoliveira@unipampa.edu.br (M.T.d.O.)

**Keywords:** NSAIDs, dogs, nanocapsules, population pharmacokinetic modeling

## Abstract

**Background/Objectives**: Meloxicam (MLX) is a nonsteroidal anti-inflammatory drug (NSAID) recommended for treating acute and chronic pain in dogs, frequently administered prophylactically to mitigate postoperative pain; however, its utility is limited by characteristic NSAID-associated adverse effects, such as gastrointestinal side effects. Nanosystems offer the potential to minimize adverse effects by sustaining drug release. Therefore, this study assessed the pharmacokinetics of MLX nanoencapsulation in female dogs undergoing ovariohysterectomy using a population pharmacokinetic (PopPK) modeling approach. **Methods**: MLX-loaded polymeric nanocapsules (NC-MLX) were prepared using the nanoprecipitation method and characterized by zeta potential, pH, mean diameter, particle size distribution, and drug content. Dogs received 0.2 mg/kg of either NC-MLX or free MLX orally, 4 h before surgery, and plasma samples were analyzed using an HPLC-PDA method. Pharmacokinetics were characterized by non-compartmental analysis and PopPK modeling. Several compartmental structures, variability models, and residual error models were explored, and relevant covariates were investigated. **Results:** NC-MLX had an average diameter of 326 ± 13 nm, a zeta potential of −26.2 ± 6.4 mV, and drug loading of 99.47% ± 0.01%. NC-MLX showed a significant increase in the t_1/2_ (36.99 ± 17.26 h) of MLX compared to the free drug (15.22 ± 4.4 h). The best-fitting PopPK model was a two-compartment model with double extravascular first-order absorption rate constants (Ka_1_ and Ka_2_), including a lag time for Ka_2_ and linear elimination, describing the second peak observed in several animals. The nanoformulation was a significant covariate for Tlag_2_, delaying the time for absorption (1.22 and 2.55 h for free MLX and NC-MLX, respectively) and increasing V_2_ (0.134 and 0.402 L/kg for free MLX and NC-MLX, respectively). External model validation showed that the final PopPK model accurately predicted plasma concentrations, with MPE% and RMSE values below 15%. **Conclusions**: Our findings suggest that NC-MLX alters MLX absorption and distribution profiles, supporting its potential as an alternative for postoperative pain management in dogs.

## 1. Introduction

Nonsteroidal anti-inflammatory drugs (NSAIDs) are routinely prescribed by veterinarians for musculoskeletal pain, arthritis, and chronic pain, as well as for postoperative pain management, reducing the need for rescue analgesia [1,2,3]. Ovariohysterectomy remains the most common surgical procedure in clinical veterinary medicine, making pain management a priority for animal welfare [4]. Meloxicam (MLX), which inhibits prostaglandin synthesis via the COX-2 pathway, has been reported as a promising alternative for pain relief [5,6].

Despite their efficacy, NSAIDs in dogs are constrained by gastrointestinal adverse effects, ranging from vomiting and diarrhea to ulceration and bleeding. These reactions are mediated by both topical irritation of the gastric mucosa and systemic COX inhibition [7,8]. Conventional oral formulations expose the stomach to high concentrations of free drugs, especially near peak absorption, narrowing the safety margin for dogs requiring prolonged therapy [9]. A clinical study with dogs reported colonic perforation and intestinal bleeding after MLX administration, revealing that extended treatment durations should be avoided [10]. In this context, nanoencapsulation represents a promising approach to uncouple analgesic efficacy from local toxicity. By entrapping the NSAID within polymeric or lipid nanocapsules, direct contact between the drug and the gastric mucosa can be minimized, the site and rate of release along the intestine can be modulated, and absorption efficiency can be enhanced, thereby improving pharmacokinetics and pharmacodynamics [7,11]. This approach is particularly appealing in veterinary medicine, where safer NSAID formulations could reduce gastrointestinal side effects and facilitate the broader use of long-term analgesia in dogs that are currently undertreated or considered high risk.

Our research group recently evaluated MLX-loaded polymeric nanocapsules (NC-MLX) to enhance their performance in the central nervous system [12,13,14]. Nakama and collaborators [14] developed NC-MLX and demonstrated their safety in rodents. Building on this, we assessed the efficacy of co-encapsulating MLX and curcumin to treat neuroinflammation in a mouse model of Alzheimer’s disease, which improved drug delivery to the brain and modulated inflammation, thereby providing neuroprotective effects [13]. Since the formulation was established as safe in rodents, its analgesic effect was subsequently evaluated in a prospective, randomized clinical trial involving female dogs undergoing ovariohysterectomy. These clinical findings demonstrated that NC-MLX provides effective postoperative analgesia in female dogs undergoing ovariohysterectomy, with fewer gastrointestinal adverse effects compared to free MLX [15].

In recent years, pharmaceutical companies specializing in veterinary medicine have shown growing interest in developing drug delivery systems for animal populations, aiming to improve pharmacokinetic and pharmacodynamic performance to promote greater animal welfare [16]. Population pharmacokinetic (PopPK) modeling has become a valuable tool in veterinary medicine, enabling researchers and industry professionals to quantify variability among individuals and breeds [17]. Given this scenario, we sought to evaluate the influence of nanoencapsulation on the pharmacokinetics of MLX in female dogs undergoing ovariohysterectomy using a PopPK approach.

## 2. Results

### 2.1. Nanocapsules Characterization

The formulations exhibited a milky, opalescent white/blue hue and were free of any precipitates. NC-MLX exhibited a nanoscale particle size of 326 ± 13 nm and a negative zeta potential (−26.2 ± 6.4 mV), both of which are typical for this type of formulation. The NC-MLX showed a slightly acidic pH (6.15 ± 0.04) and a SPAN value below 2 (1.10 ± 0.23), indicating a uniform particle size distribution. The drug content and encapsulation rate were 99.47% ± 0.01% and 99.71% ± 0.03%, respectively.

### 2.2. Non-Compartmental Pharmacokinetic Analysis

Plasma concentration–time profiles are shown in the Supplementary Material (Figure S2). The mean non-compartmental pharmacokinetic parameters for the free MLX and NC-MLX groups are listed in Table 1. Following NC-MLX administration, T_max_ increased 2.4-fold compared to the free drug (1.8 ± 0.4 and 4.37 ± 1.36 h for free MLX and NC-MLX, respectively). A slight increase in NC-MLX bioavailability was reflected in the tendency for Vd/F to increase from 0.3 ± 0.1 to 0.7 ± 0.5 L/kg, while the plasma half-life was extended by 2.4 times (from 15.22 ± 4.4 to 36.99 ± 17.26 h). No significant differences were observed in the other parameters.

### 2.3. PopPK Modeling

**Figure 1:**
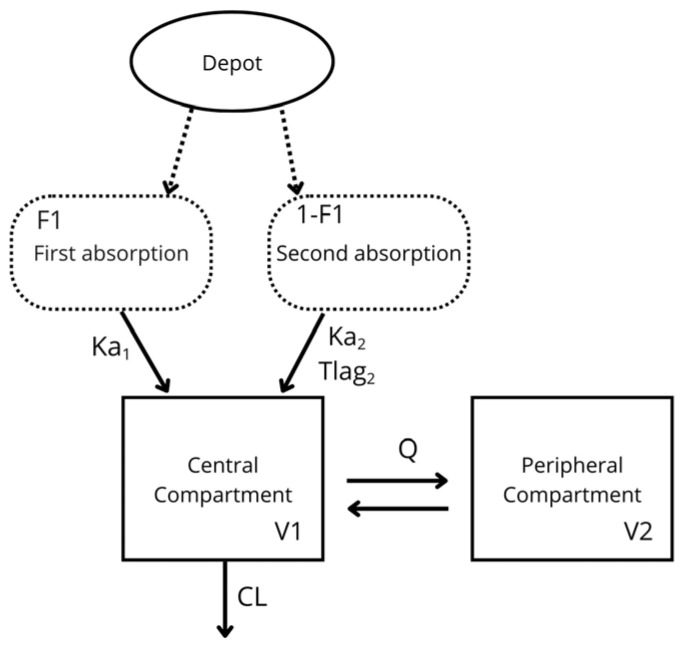
Final model structure for MLX pharmacokinetics following oral administration in female dogs. A two-compartment pharmacokinetic model, with double extravascular absorption composed of a first-order absorption process (ka_1_) and a simultaneous first-order absorption process (ka_2_) with a lag time, best described the pharmacokinetics of MLX in plasma. The dashed arrow represents the drug’s passage from the depot compartment to the transit compartments. The solid arrow represents the drug’s distribution among the main compartments. Ka_1_: first-order absorption rate constant; F1: absorbed fraction in the first absorption; Ka_2_: first-order absorption; 1-F1: absorbed fraction in the second absorption; Tlag_2_: lag time in the second absorption; V_1_: central volume of distribution; Q: intercompartmental clearance; V_2_: peripheral volume of distribution; CL: systemic clearance of MLX.

The final PopPK model describing plasma concentration versus time was a two-compartment pharmacokinetic model with double extravascular absorption: one first-order absorption (ka_1_), a simultaneous first-order absorption (ka_2_) with a lag time, and linear elimination (Figure 1). The final parameters are shown in Table 2. The residual error was described as proportional.

Interindividual variability was maintained for all PK parameters except Q. Formulation was included as a covariate for the lag time at ka_2_ (Tlag_2_pop_Free MLX_ = 1.22 h; Tlag_2_pop_NC-MLX_ = 2.55 h), representing a twofold increase, and for the volume of the peripheral compartment (V_2__pop), showing a threefold difference (NC-MLX = 0.406 L/kg; free MLX = 0.134 L/kg). Including formulation as a covariate in the model reduced the likelihood value. A decision tree representation generated by Sycomore^®^ for the final model is presented in the Supplementary Material (Figure S3), along with the Objective Function Value (OFV) (Table S2).

#### Internal and External Evaluation of the PopPK Model

**Figure 2:**
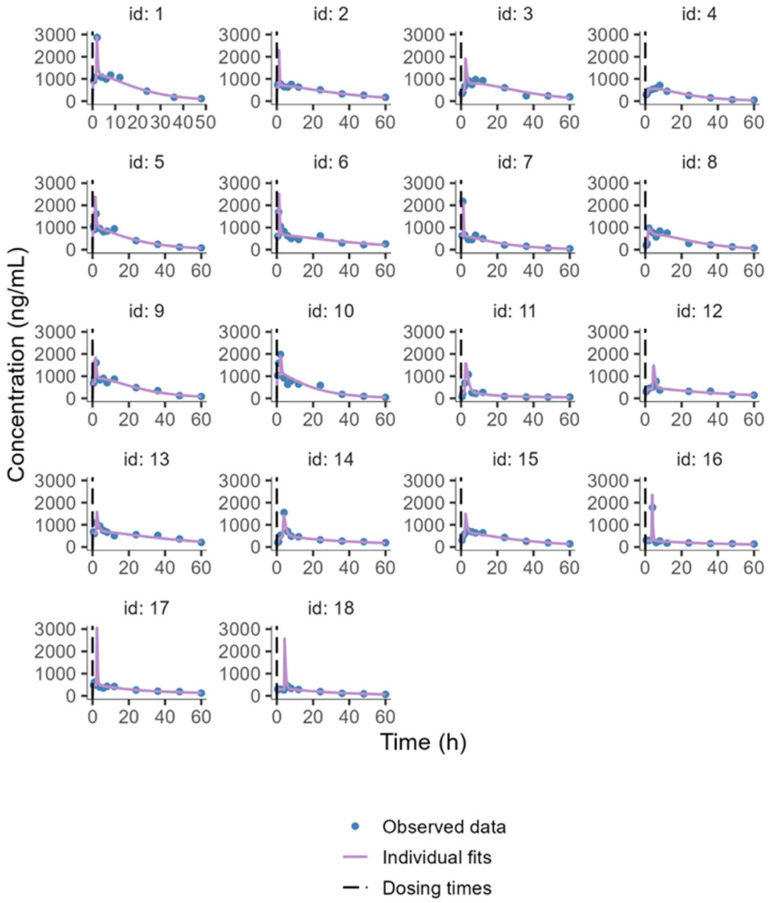
Individual concentration-time profiles after oral administration of MLX (0.2 mg/kg). Individual 1–9 (free MLX); 10–18 (NC-MLX). Dots represent the observed data. Solid lines represent the predicted value. Dashed lines represent the dosing time.

**Figure 3:**
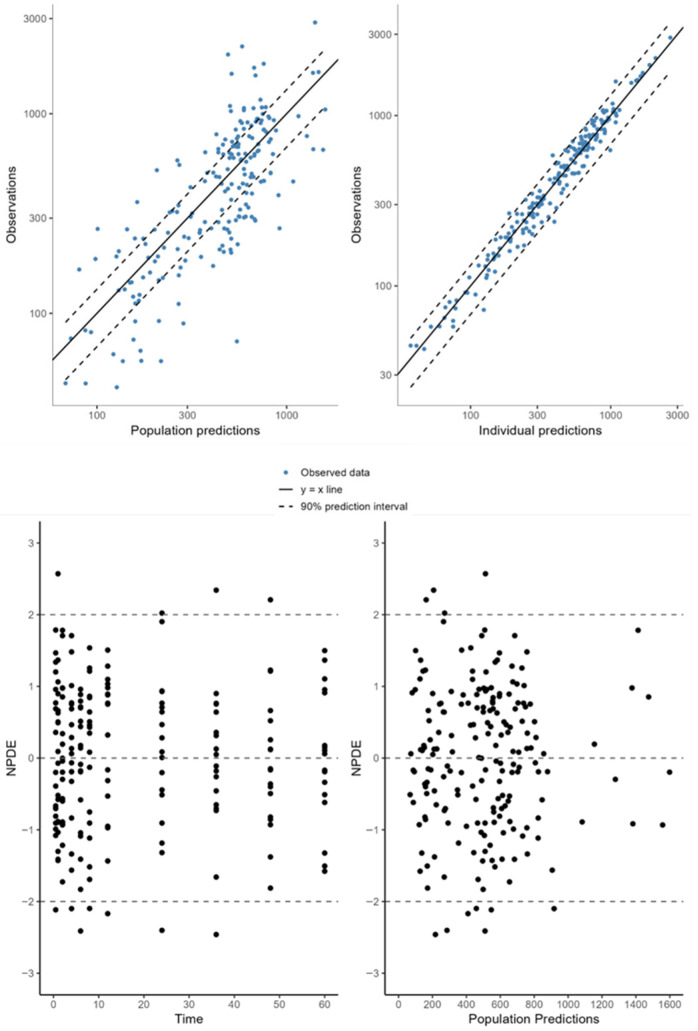
Diagnostic plots for the MLX PopPK model. Top panel: Observation vs. individual predictions plot. The solid lines indicate the line of identity; dashed lines indicate the 90% prediction interval. Each point corresponds to an observed concentration and its respective model prediction. Bottom panel: Residual plot for the MLX pharmacokinetic model. Each point corresponds to a single observation; residuals are expected to be randomly scattered around zero.

**Figure 4:**
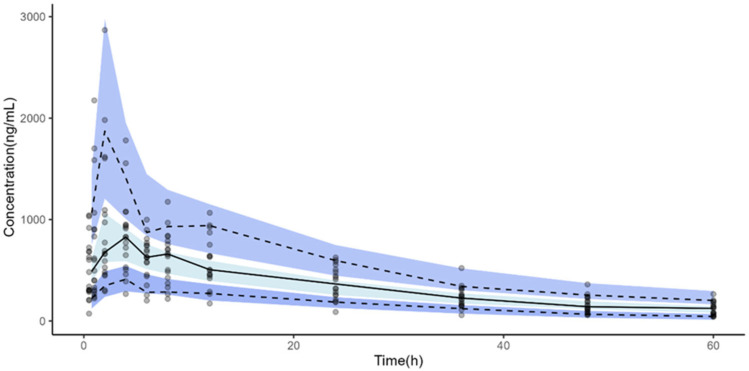
Visual predictive check of the PopPK model after single oral administration of MLX, with a 90% prediction interval. The 10th, 50th, and 90th empirical percentiles of the observed data are also demonstrated. Dark blue represents the 10th and 90th empirical percentiles. Light blue represents 50th empirical percentiles. Solid lines indicate empirical percentiles, while dashed lines represent predicted percentiles. Dots represent the observed data points. A total of 1000 datasets were simulated using the final pharmacokinetic parameter estimates.

The individual animal PK profiles and prediction range, from 1 to 9 for the free MLX group and from 10 to 18 for the NC-MLX group, are illustrated in Figure 2. Plots of observed versus individual or population-predicted values showed a good model fit, as evidenced by the goodness-of-fit plots in Figure 3, which demonstrate adequate linearity between observed and predicted concentrations. The balanced distribution of points around the zero line in the residual plots (Normalized prediction distribution errors) versus time and predicted population concentrations (Figure 3, bottom panel) further suggests that the selected proportional error model in the final analysis appropriately described the drug’s unexplained residual variability. The individual weighted residuals were randomly distributed around zero and remained within the expected limits. Figure 4 displays the visual predictive check plots based on 1000 simulations from the final model. The predicted values generated by the final PK model closely matched the observed median, confirming that the model adequately described the data in both groups.

The external validation for Dataset (1) showed a mean prediction error (MPE) of 1.01%. Dataset (2) comprised 66 observations from five pharmacokinetic studies of MLX in dogs and had the lowest MPE of 0.63%. The highest root mean square prediction error (RMSE) (7.04) was observed for Dataset (1), which included only 28 observations, whereas Dataset (2) exhibited a lower RMSE of 6.05%.

## 3. Discussion

We assessed the potential of MLX-loaded polymeric nanocapsules as an analgesic adjuvant in female dogs undergoing ovariohysterectomy. Nanocapsules are a potent approach to reducing undesired effects and enhancing drug efficacy, motivating their use in this study. This rationale aligns with previous clinical evidence from Lugoch et al. [15], where nanoencapsulated MLX provided effective perioperative analgesia with fewer gastrointestinal adverse effects compared to free MLX in female dogs following ovariohysterectomy. Although analgesic outcomes were not directly assessed in the present work, these prior findings support the translational relevance of investigating the pharmacokinetics of this nanoformulation. In the present study, nanocapsules improved MLX distribution in female dogs, further reinforcing their potential as a therapeutic strategy for animals undergoing ovariohysterectomy.

Applying PopPK modeling allows for more precise and reliable evaluation of biological systems by accounting for interindividual variability and assessing covariate effects on pharmacokinetic parameters [17,18,19]. Notably, there is still a lack of pharmacokinetic data, as only a few studies have been reported for dogs [20,21,22,23,24]. In fact, MLX PopPK modeling for canine subjects is scarce, and the only studies we found were for cats [25,26], ferrets [27], and flamingos [28]. Hence, to our knowledge, this is the first report of PopPK modeling of NC-MLX, offering new insight into the plasma pharmacokinetics of nanoparticulate systems in female dogs.

Among the parameters evaluated during NC-MLX characterization [12], particle size is crucial for the biopharmaceutical applicability of the nanosystem [29]. The NC-MLX produced in this study had an average diameter of 326 ± 13 nm, which is consistent with our previous study (312 ± 5.5 nm) that produced a similar MLX nanosystem [13]. A SPAN value below 2 indicates uniform diameter distribution of the nanocapsules, which is considered appropriate for polymer-based nanoparticles [29]. The negative zeta potential observed is characteristic of formulations utilizing poly (Ɛ-caprolactone), an anionic polymer [12]. These findings are comparable to another investigation conducted by our group, in which curcumin encapsulated with MLX exhibited diameters of 315 ± 6 nm and a negative zeta potential [14].

The pharmacokinetic parameters obtained for the free drug were similar to those reported elsewhere for dogs (Table 1) [22,24], ferrets, and monkeys [27,30]. Nevertheless, these studies used standardized experimental groups of specific breeds, whereas we included female mixed-breed dogs. The Fédération Cynologique Internationale recognizes over 300 dog breeds worldwide [31], and interbreeding among breeds yields a great variety of mixed-breed dogs. These mixed breeds result from diverse genetic backgrounds, which can significantly influence drug metabolism [32].

Oral absorption is increasingly recognized as a complex, multifactorial process. Studies with dogs [20,22,23,33], sea turtles [34], and sea lions [35] have reported a secondary peak in MLX plasma concentrations, similar to findings in humans [36,37], typically occurring 1 to 24 h post-administration. This biphasic absorption pattern may result from gastrointestinal recycling or enterohepatic circulation; however, without confirmatory testing, we could not determine the underlying mechanism in dogs. This phenomenon was also observed in some animals in our study, albeit those that received NC-MLX showed a lower incidence and reduced fluctuation between concentrations and peak due to the more controlled release of the drug [38,39].

Administration of the nanoparticulate system significantly altered the MLX PK profile (0.2 mg/kg) in female dogs, as evidenced by the non-compartmental results, which demonstrated increased Vd/F and a two-fold increase in t_1/2_ for NC-MLX compared with the free drug (Table 1). This is particularly promising, given that ovariohysterectomy has been shown to alter MLX distribution, leading to reductions in Vd and CL [21]. Moreover, drugs with short half-lives require frequent dosing to maintain therapeutic efficacy [40]. Therefore, the newly proposed formulation is promising, as it can reduce the required dosing frequency, decrease animal handling and associated stress, preserve postoperative analgesic efficacy, and accelerate recovery.

Our research group previously found that polysorbate-coated polymeric nanocapsules, which are comparable to the system developed in this study, also increased Vd and t_1/2_ of other drugs [41,42]. This effect is attributed to nanoencapsulated drugs remaining in circulation for extended periods, as they reduce opsonization and delay recognition by the immune system [43]. Additionally, NC-MLX significantly prolonged T_max_ (1.83 ± 0.4 h for free MLX and 4.37 ± 1.6 h for NC-MLX), with only a slight reduction in C_max_. In terms of C_max_ and T_max_, extended-release tablets containing MLX exhibited behavior similar to that observed in our study compared to the reference formulation [23].

The final PopPK model successfully fits the observed data for both NC-MLX and free MLX plasma concentrations in female dogs, providing reasonable PK estimates. Among the models tested, the two-compartment model best described MLX distribution (Figure 1). A previous cat model reported a two-compartment distribution and first-order absorption with a lag time for MLX [25]. In our investigation, however, the double-absorption model better captured the plasma concentration-time profiles of free and nanoencapsulated MLX, particularly during the absorption phase and near peak concentrations (Figure 2). In recent years, alternative absorption models have gained prominence, as they more accurately reflect gastrointestinal physiology during oral administration, representing the complexity of oral absorption [44]. Population models offer a versatile statistical approach to quantify variability in drug disposition based on individual patient characteristics and to optimize initial dosage regimens for achieving the therapeutic window [45,46,47]. Our PopPK model demonstrated that nanocapsules effectively controlled drug release, as evidenced by the slight T_lag_ observed in animals receiving NC-MLX. This controlled release helped to suppress the appearance of a second peak in plasma concentrations.

Our findings indicated that NC-MLX regulated the oral absorption of MLX and increased its distribution in the peripheral compartment (V_2_pop_NC_ = 0.406 L/kg) compared to free MLX (V_2_pop_ = 0.134 L/kg) (Table 2). This increased distribution due to nanoencapsulation can be utilized in veterinary practice to achieve satisfactory dose adjustments, reducing the frequency of administrations and minimizing the risk of accumulation and adverse effects [21]. The significant resemblance between the observed data and population model forecasts illustrated in Figure 3 further supports the predictive quality of the model.

A PopPK model for MLX in female dogs was developed and validated both internally and externally. Internal validation was performed using bootstrap resampling with 1000 runs, which yielded estimates closely aligned with the final population model parameters and their 95% confidence intervals. External assessment is the most rigorous method for evaluating a model’s predictive ability. For external validation, two new datasets were built. The first comprises two studies with MLX liquid formulations, and the second comprises five studies with MLX oral and liquid formulations. The final parameter estimates of the PopPK model were fixed to calculate various errors, including MPE% and RMSE [48]. Additional evaluations demonstrated that our final PopPK model reliably predicted plasma concentrations of both solid and liquid MLX forms administered orally.

Some physiological factors may interfere with or alter MLX PK in the organism, as some researchers have found notable differences in the metabolism and disposition of MLX between dogs and cats, birds, and rats [22,49,50]. The sustained release of MLX from polymeric nanocapsules can prolong effective plasma concentrations, potentially reducing the need for frequent dosing during the immediate postoperative period. As a result, NC-MLX confers advantages over conventional MLX formulations by reducing administration frequency [23,51], thereby making its use a safe adjuvant treatment for perioperative analgesia in female dogs undergoing ovariohysterectomy [15].

## 4. Materials and Methods

### 4.1. Chemicals and Reagents

Meloxicam (99.0%) and piroxicam (99.0%) (internal standard [IS]) were obtained from commercial sources. Poly(ɛ-caprolactone) (PCL; Mw = 80,000 g mol^−1^), capric/caprylic triglyceride oil (MCT, liquid), sorbitan monostearate (Span^®^ 60, solid), and polysorbate 80 (P80; Tween^®^, liquid) were obtained from Sigma-Aldrich (São Paulo, Brazil). Acetonitrile was purchased from JT Baker Chemical Co. (Avantor, Shanghai, China), and purified water was prepared using a Milli-Q Plus system (Millipore, Burlington, MA, USA). Polyethylene glycol (PEG-400, liquid), acetone, ethanol, o-phosphoric acid, and triethylamine were all of pharmaceutical grade.

### 4.2. Formulation Preparation

NC-MLX were prepared by interfacial polymer deposition [14]. The organic phase comprised PCL, MCT, Span 60, ethanol, and MLX (1 mg/mL), all dissolved in acetone. Following dissolution, this phase was added to an aqueous phase containing distilled water and P80. Acetone, ethanol, and a portion of the water were then removed by evaporation under reduced pressure to the desired volume. Free MLX solution (0.5 mg/mL) was prepared by dissolving MLX in PEG-400 (60% *v*/*v*) at 37 °C. After complete solubilization, water was added, and the mixture was stirred for 10 min.

### 4.3. Nanocapsule Characterization

The mean diameter and particle size distribution (SPAN) of the nanocapsules were determined by laser diffractometry. In this procedure, 200 μL of the sample was diluted in 100 mL of distilled water in the instrument sampler (Mastersizer 2000, Malvern Instruments, Malvern, UK). Zeta potential was determined by electrophoretic migration using a NanoBrook 90Plus instrument (Brookhaven Instruments, Nashua, NH, USA), with samples prepared by 1:1000 dilution in filtered 1 mM sodium chloride solution. The pH of NC-MLX was measured using a calibrated potentiometer (Hanna Instruments, Barueri, Brazil). Drug content and encapsulation efficiency were assessed by High-performance liquid chromatography coupled to a diode array detector (HPLC-PDA) (Shimadzu, Kyoto, Japan), as previously described [12,52].

### 4.4. Experimental Design

Eighteen healthy female dogs (*n* = 9/group) of different breeds aged 9–48 months (10.5–16.6 kg) were recruited. Animals were hospitalized 24 h before surgery for acclimatization to the experimental environment. They were sourced from the UNIPAMPA Veterinary Hospital after guardians provided written consent. Demographic data and operating procedures are included in the Supplementary Material (Table S1).

Each dog received an oral dose of either NC-MLX (0.2 mg/kg; 2.8 ± 0.3 mL) or free MLX (5.2 ± 0.4 mL) 4 h before ovariohysterectomy. Approximately 3 mL of blood was collected via the jugular vein into heparinized tubes at the following post-dose times: 0.5, 1, 2, 4, 6, 8, 12, 24, 36, 48, and 60 h. For plasma separation, samples were centrifuged at 12,000 rpm for 10 min at 4 °C; plasma was transferred to 2 mL microtubes and stored at −80 °C until analysis.

### 4.5. Meloxicam Quantification in Plasma

Plasma MLX concentrations were determined using a previously validated HPLC-PDA method. Analyses were performed on a Shimadzu LC system with an LC-20AT pump, SPD-M20A photodiode array detector, CBM-20A system controller, DGU-20A3 degasser, and SIL-20A autosampler. To each 250 µL plasma sample, 25 µL of piroxicam (IS, 100 ng/mL) was added, mixed, followed by 1000 µL acetonitrile (extraction solvent). Samples were vortexed for 5 min and centrifuged at 12,000 rpm for 10 min at 4 °C. The supernatant was transferred to a glass tube and evaporated under a gentle nitrogen stream at 60 °C. The residue was reconstituted with 100 µL acetonitrile.

Separation was achieved on a Sunfire C18 column (150 × 4.6 mm, 5 µm; Waters, Beverley, MA, USA) with a guard column (4 × 3 mm ID) packed with the same material. Chromatographic analyses were performed at 25 ± 1 °C using a mobile phase of water:acetonitrile:triethylamine (50:50:0.05, *v*/*v*/*v*), adjusted to pH 3 with orthophosphoric acid. The mobile phase was filtered through a 0.45 µm membrane (Millipore, St. Louis, MO, USA) and degassed ultrasonically before use. Flow rate was 1 mL/min. Detection occurred at 355 nm after injecting 20 µL of the sample.

### 4.6. Pharmacokinetic Analysis

#### 4.6.1. Non-Compartmental Analysis

Non-compartmental analysis was conducted using PKanalix^TM^ (v. 2021R2, Lixoft, Lancaster, California, USA) to evaluate plasma drug concentration-time profiles. Parameters calculated included area under the curve (AUC) from time zero to infinity (AUC_0–∞_), AUC from zero to time t (AUC_0–t_) the percentage of extrapolated AUC from the last measurable time point to infinity (%AUC_ext_), maximum plasma concentration (C_max_), time to reach the maximum plasma concentration (T_max_), mean residence time, clearance (CL/F), distribution volume (Vd/F), terminal plasma half-life (t_1/2_), and relative bioavailability (F_rel_) [53]. Absolute bioavailability was determined using intravenous data reported by Karademir et al. [21]. Results are expressed as mean ± standard deviation. Statistical comparisons between groups used a parametric *t*-test; *p* < 0.05 was considered significant.

#### 4.6.2. Population Pharmacokinetic Modeling

PopPK modeling was performed using Monolix^®^ (v. 2021R2, Lixoft, Lancaster, California, USA). Individual plasma concentration-time profiles were analyzed using the stochastic approximation expectation-maximization algorithm for nonlinear mixed-effects models. A total of 196 plasma concentration observations from 18 female dogs (*n* = 9/group) were included in the PopPK dataset. All values were above the quantification limit. The structural model was built by comparing one-, two-, or three-compartment models.

Residual variability was evaluated using additive, proportional, and combined error models. Interindividual variability was modeled on fixed-effect parameters using an exponential model (Equation (1)):

(1) $$Pi\;=\;Ppop.\exp\left(\eta i,P\right)$$

where Pi is the estimate for the individual lognormally distributed parameter, Ppop is the typical parameter estimate for the population, and ηi,P is the random effect accounting for individual differences from the typical value, assumed to be normally distributed with mean zero and variance Ω.

Covariate effects (e.g., type of formulation, age, and weight) were investigated using a forward inclusion approach followed by backward elimination. The covariate model was selected according to the highest likelihood and changes in interindividual and residual variability. Model outputs and comparisons between models were evaluated using Monolix^®^ and Sycomore^®^ (v. 2021R2, Lixoft).

#### 4.6.3. Internal Evaluation

Model adequacy was assessed through visual inspection of goodness-of-fit plots generated by Monolix^®^. This evaluation included observed versus individual predicted values, as well as plots of individual weighted residuals versus time and versus concentration. Analytical certainty was determined by evaluating the precision of parameter estimates using relative standard errors, alongside estimates, changes in Akaike information criterion, log-likelihood (-2LL), visual predictive checks, and their prediction intervals (5th and 95th percentiles). Confidence intervals for the parameters were obtained using a nonparametric bootstrap procedure with 1000 replicates, which also evaluated the robustness of the final model. Bootstrapping for the final PopPK model was conducted with the Rsmlx R package (v. 4.3.0, R Foundation for Statistical Computing).

#### 4.6.4. External Predictive Performance of PopPK Model

To evaluate the predictive performance and reliability of the model, external validation was performed using an independent dataset containing the following disposition data:

Dataset (1): This dataset comprises studies involving liquid formulations used to evaluate the PK of MLX administered orally in male and female dogs [22,23].
Dataset (2): This dataset includes studies that examined both solid and liquid formulations to evaluate the PK of MLX administered orally in male and female dogs [20,21,22,23,24].

Dataset (1) contained a total of 28 plasma observations, and Dataset (2) contained 66 plasma observations; both datasets were derived from the mean plasma profiles reported in the corresponding studies.

Predictions for the new datasets were obtained using Monolix^®^ with fixed values from the final model. The accuracy and precision of the model predictions were evaluated based on observed (C_obs_) and predicted (C_pred_) concentrations, using MPE and RMSE, according to Equations (2) and (3), respectively [48,54,55]:

(2) $$MPE\;\left(\%\right)=\;\frac{1}{N}\sum \frac{Cpred-Cobs}{Cobs}\;\times \;100$$

(3) $$RMSE=\sqrt{\frac{1}{N}\sum {\left(\frac{Cpred-Cobs}{Cobs}\right)}^{2}}\times 100$$

## 5. Conclusions

Our study demonstrated the pharmacological potential of NC-MLX, characterized by favorable pharmaceutical properties (mean diameter < 330 nm, zeta potential −26.2 ± 6.4 mV, and drug loading near 100%) and significant alterations in the distribution of MLX compared to the free formulation. NC-MLX significantly prolonged the half-life of MLX (36.99 ± 17.26 h vs. 15.22 ± 4.40 h for free MLX) and increased the peripheral volume of distribution (V_2_: 0.402 vs. 0.134 L/kg), resulting in a more sustained and modulated exposure profile. The final PopPK model demonstrated high accuracy and robustness, with external validation metrics (MPE% and RMSE) below 15%, indicating reliable prediction of plasma concentrations. Notably, this is the first PopPK model of NC-MLX in female dogs undergoing ovariohysterectomy, providing a quantitative framework for optimizing dosing regimens. Collectively, these findings suggest that NC-MLX is a promising therapeutic alternative for postoperative analgesia in dogs, with a pharmacokinetic profile that may reduce the gastrointestinal risk associated with NSAIDs and inform the future development of nanoformulations in veterinary medicine.

## Figures and Tables

**Table 1:** Non-compartmental pharmacokinetic analysis of meloxicam (0.2 mg/kg) orally administered in female dogs.

Parameters	Free MLX	NC-MLX
AUC_0–inf_ (µg h/mL)	27.73 ± 10.85	32.08 ± 11.7
AUC_0–t_ (µg h/mL)	23.59 ± 5.53	19.87 ± 6.49
AUC_ext_ (%)	7.66 ± 4.83	17.39 ± 8.04
C_max_ (µg/mL)	1.33 ± 0.8	0.79 ± 0.4
T_max_ (h)	1.83 ± 0.4	4.37 ± 1.6 *
t_1/2_ (h)	15.22 ± 4.4	36.99 ± 17.26 *
CL/F (mL/min/kg)	0.1 ± 0.04	0.12 ± 0.05
MRT (h)	18.61 ± 3.3	22.7 ± 4.7
Vd/F (L/kg)	0.3 ± 0.1	0.7 ± 0.5 *
F	0.94	1.09
F_rel_		1.16

AUC_0–inf_: Area under the curve from time zero to infinity; AUC_0–t_: area under the curve from time zero to the last measurable sample; AUC_ext_: percentage of the extrapolated area under the curve from the last measurable sample to infinity; C_max_: maximum plasma concentration; T_max_: time to reach maximum concentration; MRT: mean residence time; Cl/F: clearance; Vd/F: apparent volume of distribution; t_1/2_: terminal half-life; F: bioavailability; F_rel_: relative bioavailability. * Statistical difference (*p* < 0.05) detected by the *t*-test compared to the free MLX group (α = 0.05). Data is represented as mean ± SD.

**Table 2:** Estimation of the PopPK model parameters.

Parameters	Estimate (RSE%)	Covariate Effect in the Parameter	Shrinkage (%)	Bootstrap		
				Median	5th Percentile	95th Percentile
Ka_1__pop (h^−1^)	0.086 (20.1)	NC-MLX controlled MLX absorption time	6.59	0.099	0.049	0.397
Ka_2__pop (h)	1.82 (51.3)		13.5	2.722	0.626	16.602
F1_pop (h^−1^)	0.85 (5.87)		−11.6	0.81	0.652	0.944
Tlag_2__pop	1.22 (16.3)		−6.09	1.125	0.001	1.809
β_Tlag_2___NC-MLX_	0.74 (28.7)		-	0.926	−0.782	7.591
					
CL_pop (mL/min)	0.1 (8.56)		6.52	0.099	0.087	0.121
V_1__pop (L/kg)	0.049 (56.0)		4.22	0.151	0.023	0.493
Q_pop (L/h)	0.24 (24.1)		-	0.271	0.09	1.046
V_2__pop (L/kg)	0.134 (27.8)		−5.93	1.394	0.296	2.207
β_V_2___NC-MLX_	1.11 (31.4)	NC-MLX increased V_2_ values		1.081	0.229	2.836
					
IIV						
ωKa_1_ (%)	0.38 (21.1)			0.274	0.042	0.577
ωKa_2_ (%)	1.28 (35.3)			0.490	0.137	5.683
ωF1 (%)	0.67 (53.8)			0.757	0.146	1.852
ωTlag_2_ (%)	0.38 (20.7)			0.428	0.112	3.964
ωCL (%)	0.32 (19.4)			0.253	0.054	0.427
ωV_1_ (%)	1.44 (29.8)			0.810	0.079	1.804
ωV_2_ (%)	0.65 (20.3)			0.597	0.184	1.27
Residual variability						
Proportional model	0.19 (6.83)			0.192	0.154	0.24

Ka_1_: First-order absorption rate constant for the first absorption process; Ka_2_: first-order absorption rate constant for the second absorption process; F1: fraction absorbed in the first absorption process; Tlag_2_: lag time for the second absorption process; V_1_: central volume of distribution; Q: intercompartmental clearance; V_2_: peripheral volume of distribution; CL: systemic clearance; β: covariate effect parameter; IIV: interindividual variability; Ω: variance of the interindividual variability; σ: error model; RSE: relative standard error.

## Data Availability

The original contributions presented in this study are included in the article/supplementary material. Further inquiries can be directed to the corresponding author.

## Supplementary Materials

The following supporting information can be downloaded at https://www.mdpi.com/article/10.3390/ph19030412/s1, Table S1: Demographics, and operative (mean ± standard deviation) of female dogs premedicated with meloxicam (0.2 mg/kg) before ovariohysterectomy. Figure S1: Size distribution (A) and zeta potential (B) of particles. Figure S2: Plasma concentration–time profile after oral administration of MLX in female dogs. Table S2: Sequential structural model development. Figure S3: Model development. Figure S4: Histogram of theoretical (dotted line) and empirical (blue bars) distribution of individual-weighted residuals (IWRES), and normalized prediction distribution errors (NPDE), based on 500 simulations of the PopPK model (conditional distribution). Figure S5: Histogram of theoretical (black line) and empirical (blue bars) distribution of individual parameters, based on 500 simulations of the PopPK model. The values correspond to the shrinkage-η obtained for each IIV (conditional distribution). Table S3: Pharmacokinetic studies of MLX in dogs. References [21,22,23,24,25] are cited in the Supplementary Materials.
