# Supplementary material for: Evaluating Orally Administered Meloxicam-Loaded Polymeric Nanocapsules in Female Dogs: A Population Pharmacokinetic Modeling Study
Source: Pharmaceuticals (Basel). 2026 Mar 3;19(3):412. doi: 10.3390/ph19030412 (PMC13028730; doi:10.3390/ph19030412)
Supplement: Supplementary file 1 [file pharmaceuticals-19-00412-s001.zip › pharmaceuticals-4136069-supplementary.pdf]

Supplementary Material
Part I – General information

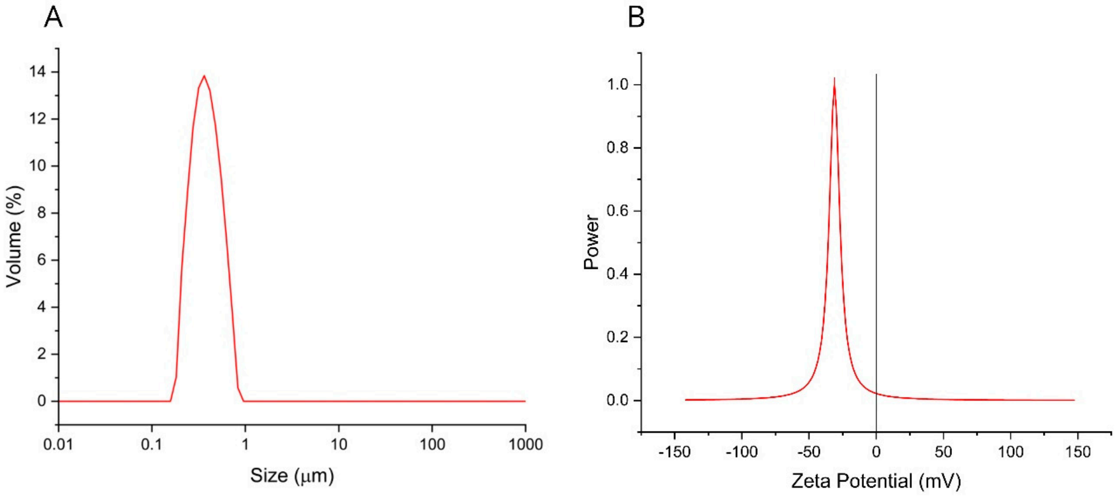

Figure S1 Size distribution (A) and zeta potential (B) of particles.

Table S1 Demographics, and operative (mean ± standard deviation) of female dogs premedicated with meloxicam (0.2 mg/kg) before ovariohysterectomy

	**Group**	
	**Free MLX**	**NC-MLX**
Age (months)	26.1 ± 18.3	19.5 ± 14.4
Body Weight (kg)	14.2 ± 1.6	12.9 ± 1.8
Total duration of anesthesia (min)	100 ± 16	109 ± 31
Total duration of surgery (min)	55 ± 21	60 ± 22

Parte II – Pharmacokinetic analysis

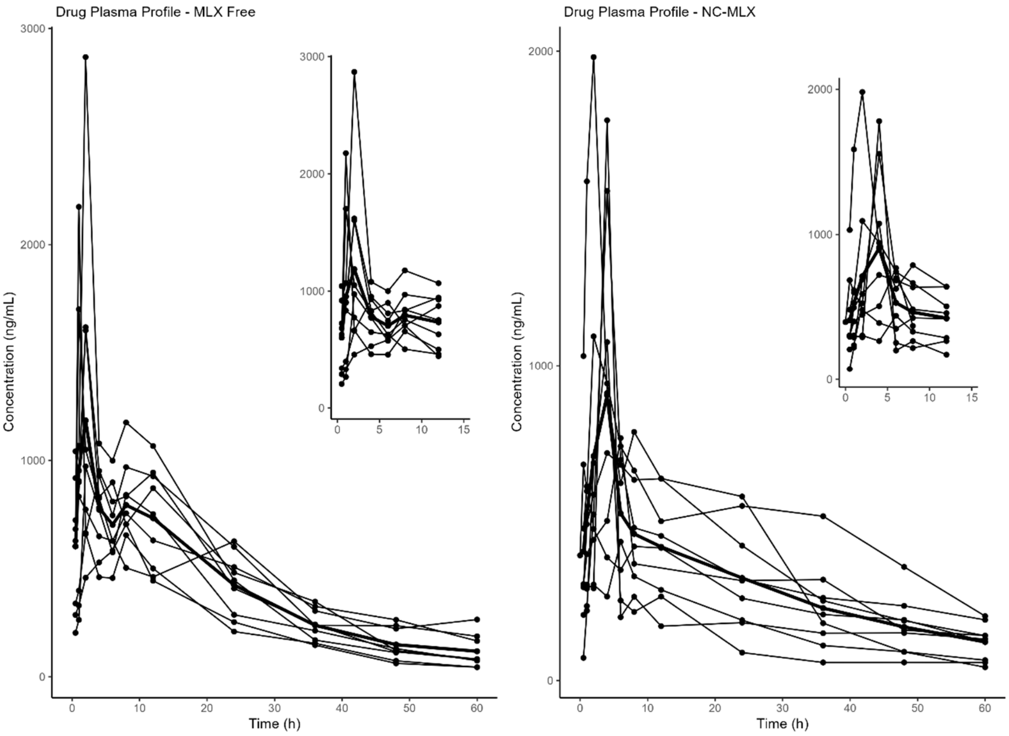

Figure S2 Plasma concentration–time profile after oral administration of MLX in female dogs. Dots and thin solid lines represent individual observed concentration-time profiles, while the thick solid line represents the mean profile. The inset provides a magnified view of the early sampling period to enhance visualization of the absorption phase.

Selection of model using Sycomore

Firstly, the structural model was developed by testing one- and two-compartment models. Next, different absorption processes were evaluated, and the models continued to be assessed by Sycomore®.

Table S2 Sequential structural model development

Model	OFV (-2LL/AIC)	
	Absence of covariates	Presence of covariates
1 compartment with first-order absorption	2577.17 / 2593.17	2551.86 / 2571.76[a,b]
2 compartments with first-order absorption	2547.61 / 2569.61	2535.16 / 2561.16[b,c]
2 compartments with first-order absorption + Tlag	2542.9 / 2568.9[c]	2531.08 / 2561.08[c]
2 compartments with transit compartments	2537.78 / 2567.78	2531.06 / 2563.06[c]
2 compartments with double extravascular absorption (first-order + simultaneous first-order absorption)	2561.72 / 2589.72	2534.76 / 2568.76[a,b,c]
2 compartments with double extravascular absorption (zero-order + sequential first-order absorption)	2555.88 / 2587.88	2538.99 / 2554.13[c]
Final model	2504.66 / 2540.66[c]	

Covariates tested: Age[a], Weight[b], Formulation[c].

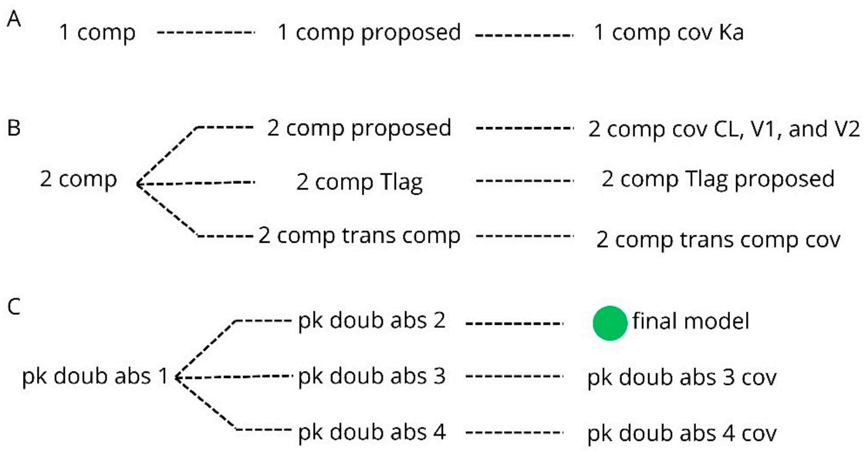

Figure S3 Model development. (A) Evaluation of a one-compartment model. (B) Evaluation of a two-compartment model. (C) Exploration of alternative absorption models.

Internal evaluation

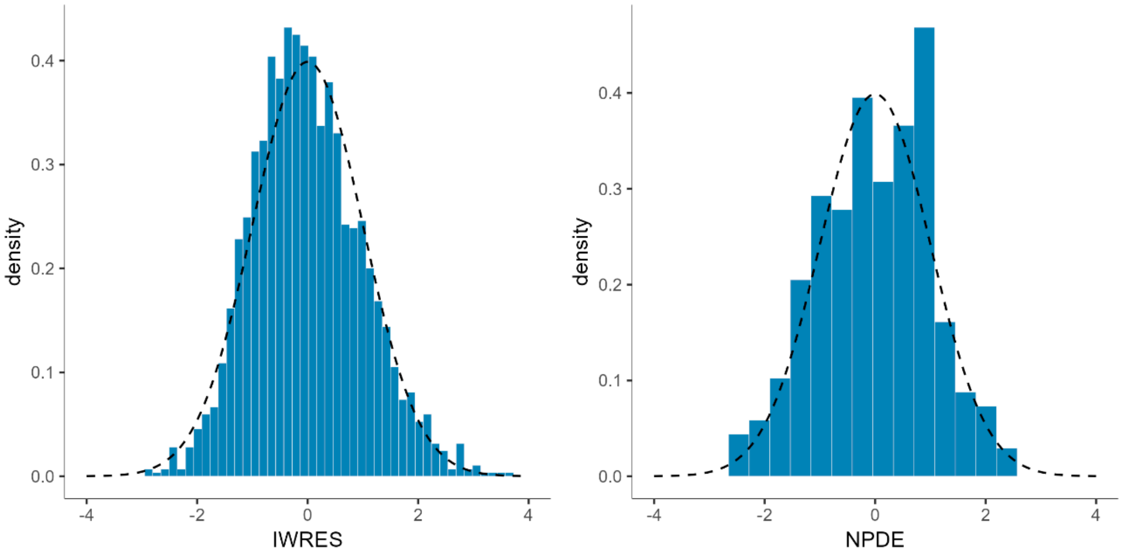

Figure S4 Histogram of theoretical (dotted line) and empirical (blue bars) distribution of individual-weighted residuals (IWRES), and normalized prediction distribution errors (NPDE), based on 500 simulations of the PopPK model (conditional distribution).

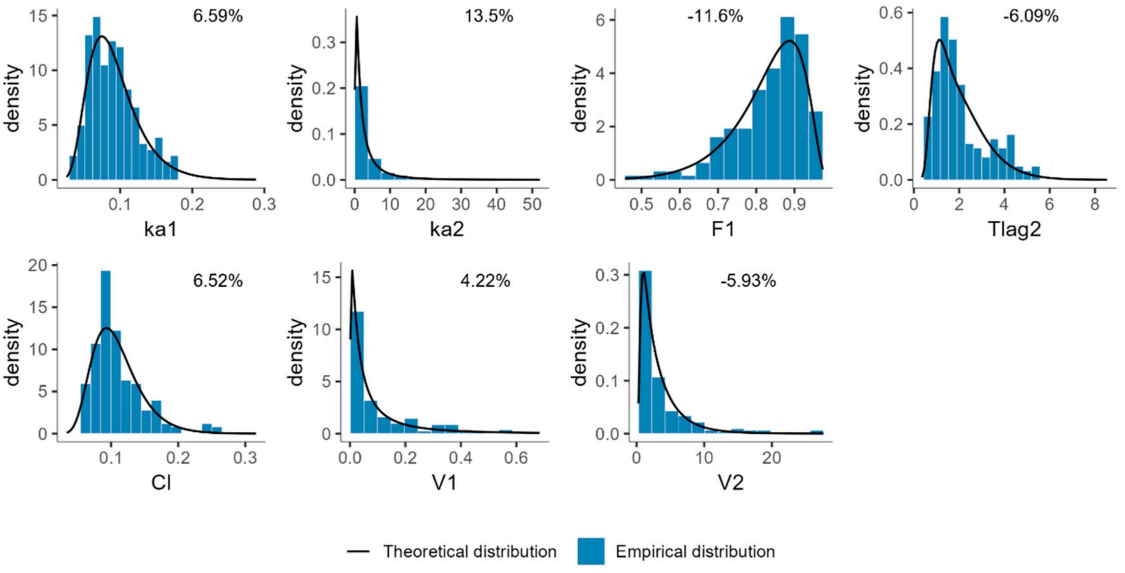

Figure S5 Histogram of theoretical (black line) and empirical (blue bars) distribution of individual parameters, based on 500 simulations of the PopPK model. The values correspond to the shrinkage-η obtained for each IIV (conditional distribution).

External evaluation

For performed external evaluation, firstly the data from five pharmacokinetic studies [1–5] of meloxicam in dogs were extracted through WebPlotDigitizer 4.6. These articles were chosen based on the dose, and species which were similar to our study (see Table S2). The data was separated into two datasets: (1) the studies that administrated suspension and (2) all five studies. Using Monolix2021R2 software (LIXOFT SAS, Simulations Plus), individual plasma concentration profile data were analyzed using the stochastic approximation expectation maximization algorithm (SAEM) for nonlinear mixed effects. A total of 28 (1) and 66 (2) plasma observations derived from the mean plasma profiles of the studies were included in the dataset for PopPK analysis. The value estimates in the PopPK final model were fixed.

The summary of five studies used in the external validation is presented in Table S3.

Table S3 Pharmacokinetic studies of MLX in dogs

Reference	Experimental Design	Main Results
Bush et al., 1998	The study was conducted with six beagle dogs (3 male and 3 female). A single oral dose of MLX (0.2 mg/kg, pellets). Blood samples were collected up to 96 hr after the dose. The MLX in plasma was quantified using HPLC.	C_{max} – 21.5 mg/L<br>T_{max} – 7.5 h<br>AUC_{0-inf} – 22.9 mg.h/L<br>MRT – 40 h<br>$t_{1/2}$ – 23.7 h<br>Vz/F – 0.3 L/kg<br>CL/F – 0.009 L/h/kg
Montoya et al., 2004	The study was conducted with eight mixed-breed dogs (4 male and 4 female). A single oral dose of MLX (0.2 mg/kg, suspension). Blood samples were collected up to 72 hr after the dose. The MLX in plasma was quantified using HPLC.	C_{max} – 0.82 μg/mL<br>T_{max} – 8.5 h<br>AUC_{0-t} – 14.61 μg.h/mL<br>$t_{1/2}$ – 12.13 h<br>Vd/F – 0.23 L/kg<br>CL/F – 10 mL/h.kg

Yuan et al., 2009	The study was conducted with six beagle dogs (3 male and 3 female). A single oral dose of MLX (0.31 mg/kg, tablet). Blood samples were collected up to 96 hr after the dose. The MLX in plasma was quantified using LC/MS/MS.	C_{max} – 17.5 ng/mL<br>T_{max} – 4 h<br>AUC_{0-t} – 23.9 μg.h/mL<br>AUC_{0-inf} – 24.8 μg.h/mL<br>MRT – 24.4 h<br>$t_{1/2}$ – 17.5 h<br>V/F – 0.32 L/kg<br>CL/F – 0.21 mL/min/kg
Karademir et al, 2016	The study was conducted with six cross-bred bitches. A single oral dose of MLX (0.2 mg/kg, tablet). Blood samples were collected up to 96 hr after the dose. The MLX in plasma was quantified using HPLC.	C_{max} – 0.39 ± 0.13 μg/mL<br>T_{max} – 5 ± 1.41 h<br>AUC_{0-inf} – 17.99 ± 4.97 μg.h/mL<br>MRT – 56.03 ± 13.62 h<br>$t_{1/2}$ – 37.91 ± 9.15 h<br>Vd/F – 0.63 ± 0.24 L/kg<br>Cl/F – 11.90 ± 3.23 ml/kg
Smith et al, 2020	The study was conducted with seven male beagle dogs. An oral dose of MLX (0.2 mg/kg, suspension). Blood samples were collected up to 72 hr after the dose. The MLX in plasma was quantified using LC-MS.	C_{max} – 295 ng/mL<br>T_{max} – 4 h

References

1. Busch, U.; Schmid, J.; Unther Heinzel, G.¨; Schmaus, H.; Urgen Baierl, J.¨; Huber, C.; Roth, W. *PHARMACOKINETICS OF MELOXICAM IN ANIMALS AND THE RELEVANCE TO HUMANS*; 1998.

2. Karademir, U.; Aksit, D.; Kum, C.; Erdogan, H.; Ucar, E.H.; Peker, C.; Gokbulut, C. The Effect of Surgery (Ovariohysterectomy) on the Plasma Disposition of Meloxicam Following Intravenous Administration in Dogs. *BMC Vet Res* **2016**, *12*, doi:10.1186/s12917-016-0659-y.

3. Montoya, L.; Ambros, L.; Kreil, V.; Bona, R.; Albarellos, G.; Hallu, R. A Pharmacokinetic Comparison of Meloxicam and Ketoprofen Following Oral Administration to Healthy Dogs. **2004**, *28*, 415–428.

4. Smith, B.J.; Kirschner, S.M.; Kendall, L. V. Pharmacokinetics of Sustained-Release, Oral, and Subcutaneous Meloxicam over 72 Hours in Male Beagle Dogs. *Journal of the American Association for Laboratory Animal Science* **2020**, *59*, 737–741, doi:10.30802/AALAS-JAALAS-19-000155.

5. Yuan, Y.; Chen, X.Y.; Li, S.M.; Wei, X.Y.; Yao, H.M.; Zhong, D.F. Pharmacokinetic Studies of Meloxicam Following Oral and Transdermal Administration in Beagle Dogs. *Acta Pharmacol Sin* **2009**, *30*, 1060–1064, doi:10.1038/aps.2009.73.
